# Non-Enzymatic Oligomerization of 3’, 5’ Cyclic AMP

**DOI:** 10.1371/journal.pone.0165723

**Published:** 2016-11-01

**Authors:** Giovanna Costanzo, Samanta Pino, Anna Maria Timperio, Judit E. Šponer, Jiří Šponer, Olga Nováková, Ondrej Šedo, Zbyněk Zdráhal, Ernesto Di Mauro

**Affiliations:** 1 Istituto di Biologia e Patologia Molecolari, CNR, Piazzale Aldo Moro, 5, Rome, 00185, Italy; 2 Dipartimento di Biologia e Biotecnologie “Charles Darwin”, “Sapienza” Università di Roma, Piazzale Aldo Moro, 5, Rome, 00185, Italy; 3 Department of Ecology and Biology, “La Tuscia” University, Viterbo, 01100, Italy; 4 Institute of Biophysics, Academy of Sciences of the Czech Republic, Královopolská 135, CZ-61265, Brno, Czech Republic; 5 CEITEC—Central European Institute of Technology, Masaryk University, Kamenice 5, CZ−62500, Brno, Czech Republic; 6 Istituto Pasteur Italia—Fondazione Cenci-Bolognetti, c/o Dipartimento di Biologia e Biotecnologie “Charles Darwin”, “Sapienza” Università di Roma, P.le Aldo Moro, 5, Rome, 00185, Italy; ENEA Centro Ricerche Casaccia, ITALY

## Abstract

Recent studies illustrate that short oligonucleotide sequences can be easily produced from nucleotide precursors in a template-free non-enzymatic way under dehydrating conditions, i.e. using essentially dry materials. Here we report that 3’,5’ cyclic AMP may also serve as a substrate of the reaction, which proceeds under moderate conditions yet with a lower efficiency than the previously reported oligomerization of 3’,5’ cyclic GMP. Optimally the oligomerization requires (i) a temperature of 80°C, (ii) a neutral to alkaline environment and (iii) a time on the order of weeks. Differences in the yield and required reaction conditions of the oligomerizations utilizing 3’,5’ cGMP and cAMP are discussed in terms of the crystal structures of the compounds. Polymerization of 3’,5’ cyclic nucleotides, whose paramount relevance in a prebiotic chemistry context has been widely accepted for decades, supports the possibility that the origin of extant genetic materials might have followed a direct uninterrupted path since its very beginning, starting from non-elaborately pre-activated monomer compounds and simple reactions.

## Introduction

Understanding the mechanisms that allowed the emergence of RNA in abiotic conditions is one of the necessary steps for the reconstruction of the processes that eventually led to life. In order to approach this problem, we have adopted a philologic approach, relying on the selection of the simplest possible materials and processes.

In this frame and moving in the bottom-up direction, we have previously reported conditions allowing the one-pot synthesis of four different nucleosides: adenosine, uridine, cytidine, thymidine[1] from a one-carbon atom molecule (formamide, NH_2_COH). The conditions for this synthesis are prebiotically plausible: the energy source is proton irradiation, mimicking solar wind; the reaction environment is the simplest possible: dryness. Nucleosides can be non-fastidiously phosphorylated ([2–4] and references therein) in different kinds of environments and are intrinsically more stable in the cyclic form than in the open form. Hence derives the possibility of their accumulation as cyclic phosphates[3] and their potential role as prebiotic precursors of ribo-oligomers.

Conditions for the polymerization of 3’,5’ cyclic GMP were explored and described in refs. [5–8], and a plausible reaction mechanism was suggested in ref. [9]. Such a template-free polymerization reaction is preceded by the self-assembling of the cyclic precursors utilizing stacking interactions, which mediate the transphosphorylations among the pillared monomer units, resulting in covalently bound oligonucleotides. An in-depth theoretical analysis of the geometrical conditions enabling the transphosphorylation between two 3’,5’ cyclic nucleotides has shown that a stacked ladder-like architecture provides with optimum steric conditions for the reaction. Indeed, the same kind of stacked architecture is found in the crystal structure of 3’,5’ cyclic GMPs.[10]

In the current work we consider another nucleotide, 3’,5’ cyclic AMP, and analyze how differences in the manner of self-assembling of the nucleotide precursors translate into differences in the oligomerization efficiency. 3’,5’ cyclic AMP appears to be a prime candidate for this purpose because its crystal geometry [11] exploits a combination of stacking and H-bonding interactions for self-organization of the nucleotides in a very peculiar manner. Here we report on the oligomerization of 3’, 5’ cyclic AMP (hereafter abbreviated as 3',5’ cAMP), with particular emphasis on the optimal conditions for the reaction, and on the characterization of the oligomerization products by MALDI ToF MS, MALDI-ToF/ToF MS, and by denaturing PAGE followed by end-labelling with ^32^P.

## Materials and Methods

### Materials

3’,5’ cAMP was obtained from BioLog LSI (Bremen, Germany) in acid form (3’,5’cAMP, H^+^) as 1 mM solution. The compound was custom made and specially purified in order to guarantee (i) the maximal possible purity relative to the absence of adducts-forming cations (mostly Na^+^) and (ii) the absence of evaporation or precipitation steps during the course of the whole process. The process by the manufacturer is described in S1 Text. The purity of the final product was 99.67% (HPLC at 253 nm), as analyzed by the Provider. MALDI ToF MS analysis showed the total absence of oligomerized materials. Doubly distilled deionized MilliQ water was used throughout. The Na-free forms were used for all the polymerization experiments. A_24_ and A_2_ were purchased from Dharmacon and Biolog respectively, and were provided in the standard lyophilized form.

### Methods

#### Polymerization of 3′,5′ cAMP

Polymerization of the cyclic nucleotides was performed as described in ref. [9]. 3’,5’cAMP Na^+^-free, H^+^ form (that was neither evaporated nor precipitated during the preparation steps) was concentrated from the initial 1mM concentration in MilliQ unbuffered water pH 7.0 by evaporation in Savant under vacuum in Eppendorf plastic tubes and cooling mode till the desired dryness was achieved. Dryness was judged from the formation of a white semi-solid aggregate. For the reaction carried out at different pH values the samples were prepared as follows. 150 μL of cyclic nucleotide solution was dried and subsequently dissolved in 150 μL of Tris HCl buffer of the desired pH. Then the sample was dried as described above. Polymerization in dry was performed by incubating the pellet obtained by evaporating typically 150 μL of 1 mM cyclic nucleotide solution (1.5x10^-7^mol) in the indicated conditions (time, temperature). The reaction was stopped by quick freezing and was followed by product analysis using MALDI-ToF MS, MALDI-ToF/ToF, or denaturing PAGE techniques. For gel electrophoresis, aliquots of the RNA samples were resuspended in 100% formamide and separated on 16% or 20% polyacrylamide gels containing 7M urea.

5’-Terminal phosphorylation of RNA oligonucleotides. See S2 Text.

#### MALDI-ToF mass spectrometry

The samples were mixed with the MALDI matrix (75 mg^.^ml^-1^ 3-hydroxypicolinic acid in water: acetonitrile 1:1, v/v mixture) and the mixture was applied onto a ground steel MALDI target. The analyses were performed with an Ultraflextreme MALDI-ToF mass spectrometer (Bruker Daltonics) in positive and in negative ion modes. The mass spectra were calibrated externally by using synthetic peptide standards and alpha-cyano-4-hydroxycinnamic acid, used also as the MALDI matrix for calibration runs. Better detection of the covalent oligomers was obtained in the negative ion detection mode.

#### MALDI-ToF/ToF mass spectrometry

The analyses were performed using the same instrument. The samples were ionized with higher laser power and the ions of interest were isolated by a precursor ion selector. After receiving additional kinetic energy induced by supplementary voltage source (LIFT), fragments formed by decay of the selected precursor ions during their flight in the TOF analyzer were separated in a reflectron.

### Experimental Results

**Fig 1:**
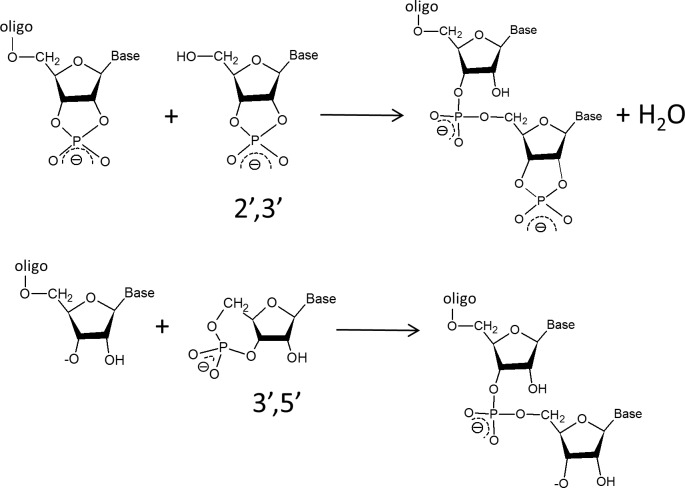
In the chain extension step of the oligomerization of 2’,3’ and 3’,5’ cyclic nucleotides an intramolecular phosphodiester linkage is replaced with an intermolecular one.

The formation of a new phosphodiester bond in extant biological RNA is based on a transesterification initiated by the nucleophilic attack of a 3’ hydroxyl group on the phosphate of the substrate. The higher is the reactivity associated with the involved leaving group, the higher is the efficiency of the polymerization reaction. When considering cyclic nucleotides as substrates, the reaction entails the concomitant opening of an intramolecular phosphodiester linkage. Basically, in the chain extension step of the oligomerization of cyclic nucleotides intramolecular phosphodiester linkages are replaced with intermolecular ones (see Fig 1).

The oligomerization of 3’, 5’ cAMP was analyzed by treating an initial 1 mM aqueous solution according to the following simple procedure: cAMP (provided by BioLog in the acid form as a 1 mM water solution at pH 7.0, completely devoid of Na^+^ and never precipitated during its preparation (see Materials), was dried (see Methods) and reacted for the indicated time periods at the indicated temperature. The reaction at different pH values was performed as described in the Methods section. The products of the reactions were analyzed by: (i) MALDI-ToF MS (from now on “MALDI” for short), (ii) by MALDI-ToF/ToF MS fragmentation (“MS/MS“), and (iii) by 5’-terminal labeling followed by denaturing PAGE.

#### MALDI ToF MS analysis of the products of the reaction

**Fig 2:**
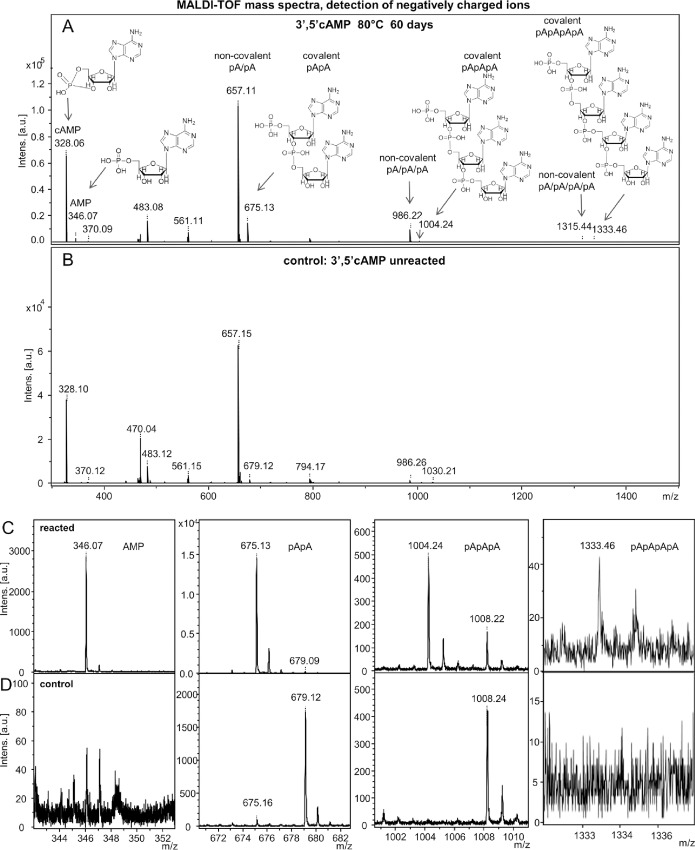
MALDI analysis of the reaction products obtained by reacting 3’,5’ cAMP for 60 days, pH 7.0, 80°C. Panel A: the overall pattern recorded for the reacted 3’,5’ cAMP sample. Panel B: spectrum of the unreacted sample. Panels C and D show the blow-ups of the relevant areas from panels A and B, respectively.

Fig 2 shows the MALDI analysis of the products obtained by treatment of 3’,5’ cAMP at 80°C, pH 7.0, for 60 days. The analysis, performed in the negative ion mode, identifies dimers, trimers and tetrames.

Fig 2, panel A shows the entire MALDI ToF mass spectra with dominating signals of non-covalent cAMP adducts. The detailed views (panels C and D) show intense signals corresponding to covalent dimer and trimer and a less intense tetramer peak (panel C, reacted). The non-reacted sample (panel D) shows only weak or no signals of these compounds. The values reported are given in the negative mode (that is: -1 relative to the actual *m/z* value). Thus: 328.06 is the 3’, 5’ cAMP; 346.07 is its open form (328+18); the series of the non-covalent gas-phase adduct forms is: 657.11 (dimer, dubbed pA/pA), 986.22 (trimer, pA/pA/pA), 1315.44 (tetramer pA/pA/pA/pA) and so on. The series of covalently bound molecules is: 675.13 (covalent dimer, dubbed pApA), 1004.24 (covalent trimer, pApApA), 1333.46 (covalent tetramer, pApApApA). The enlargements in panel C show the relevant molecules (monomer, covalent dimers, trimers and tetramers) highlighting the differences in intensity of the signals (in arbitrary units, ordinate). Panel D shows the absence of covalent oligomer signals observed in the corresponding control samples.

#### Validation by MALDI ToF/ToF

**Fig 3:**
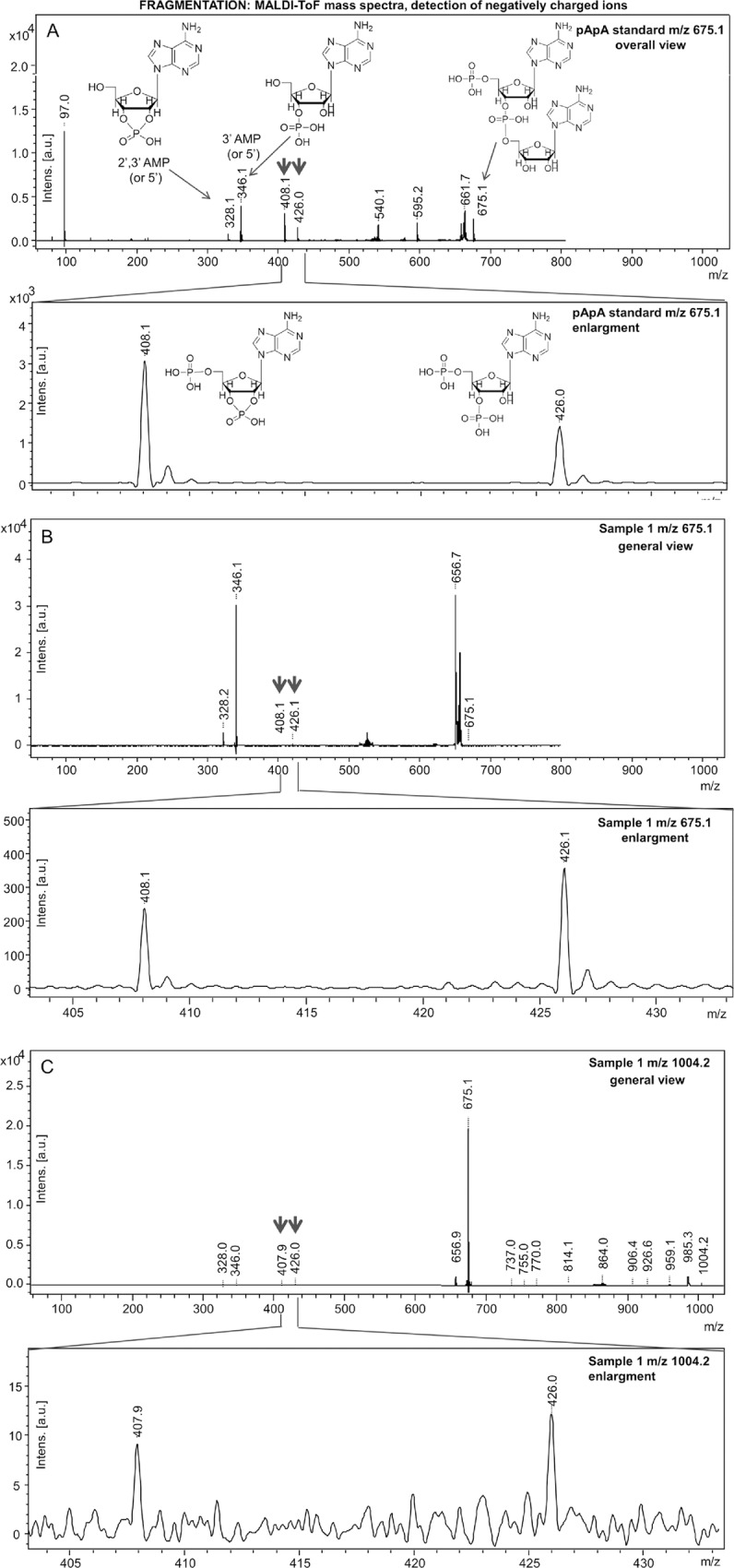
Fragmentation of the *m/z* = 675 and 1004 molecules (detected in negative ion mode), showing the presence of the species diagnostic of covalent dimers and trimers. For details, see the text. For fragmentation of the non-covalent dimer at *m/z* = 657 see S1 Fig.

The negatively charged ions with *m/z* values corresponding to the dimer and trimer (675.1 and 1004.2) were subjected to MS/MS analysis under LIFT arrangement of the instrument. Ion 675.1 obtained from a 100 μM pApA standard was also subjected to the MS/MS fragmentation under the same conditions. Fig 3 shows the spectra obtained from the standard (Panel A), as well as from the *m/z* = 675.1 (Panel B) and 1004.2 (Panel C) species, respectively. The signals that unambiguously prove formation of the covalent linkage between the nucleotides are *m/z* = 408.1 (the nucleoside bearing a phosphate at 5’ and a cyclic phosphate at the 2’ and 3’ positions, the latter hereafter abbreviated as pA>p), and *m/z* = 426.1 (the nucleoside bearing two phosphates: pA_‒_p). These signals are displayed in the corresponding blow-ups shown in Fig 3, panels A, B and C. These molecules necessarily derive from a covalent dimer, and are present in the standard and in the fragmentation profiles of both the dimers and trimers. The fragmentation of the trimer (panel C) yields, in addition to these two unambiguous indicators, also a dimer (see the signal corresponding to pApA at *m/z* = 675.1), which is compatible both with a covalent dimer (as expected from the fragmentation of a trimer) and with a non-covalent adduct of a cAMP and an AMP [12] and is thus considered not resolutory. In conclusion, in spite of the particular tendency of this system to create false-positives, the fragmentation analysis provides direct evidence of the presence of covalently-bound dimers and trimers.

**Fig 4:**
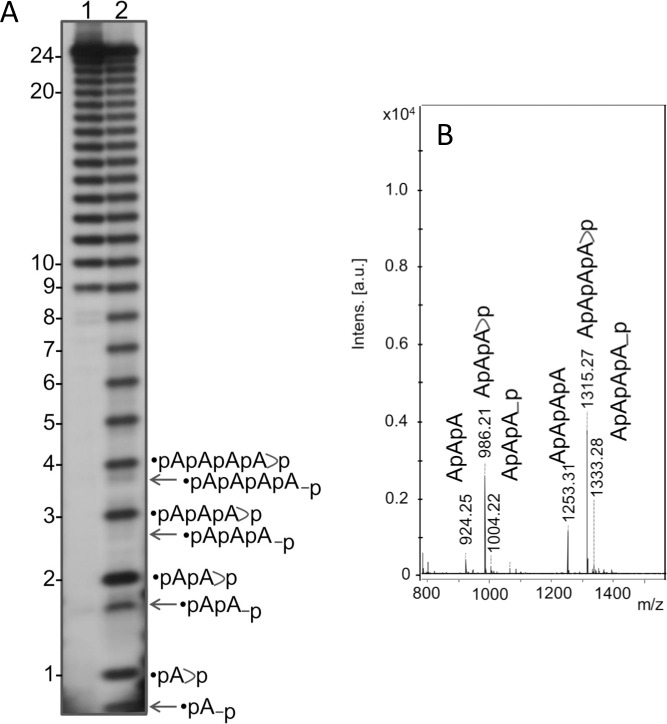
Hydrolysis of A_24_ by water or formamide. Panel A: 20% acrylamide PAGE analysis of 5’ labeled A_24_, hydrolyzed in water (Lane 1, see text) or formamide (Lane 2, see text). The product formed from A_24_ (not phosphorylated at 5’) upon digestion in formamide was analyzed by MALDI in the negative ion mode. Panel B shows, as examples, the MALDI profiles corresponding to the trimer and tetramer families produced by formamide hydrolysis. The hydrolysis produces: (i) a species with *m/z* = 986.21 corresponding to the primary product of the cleavage at 3’ = ApApA>p, (ii) a species with *m/z* = 1004.2 (986 + 18) derived from its opening to ApApA_p, (iii) a species with *m/z* = 924.25 (1004–80) derived by the phosphate-loss of this latter = ApApA. Analogous products can be derived for the tetramer: 1315.27 (ApApApA>p) opened to 1333.28 (ApApApA_p) and dephosphorylated to 1253.31 (ApApApA). The fully dephosphorylated species migrates in the gel faster than the phoshorylated ones. Likewise, in MALDI this species gives a signal at lower *m/z* value. Longer oligomers behave similarly. Thus, using a reference ladder produced this way for the interpretation of the 3’,5’ cAMP oligomerization products is fully justified. Note that in contrast to the oligomers detected by MALDI, the oligomers detected by PAGE always carry a ^32^P-phosphate group at 5’.

The MALDI analysis identifies the molecular species involved, but does not provide quantitative evaluations. In order to characterize the kinetics of the oligomerization as a function of the reaction conditions, we recurred to terminal labelling of the products, followed by high-resolution PAGE. PAGE analysis is more sensitive than MALDI, but its interpretation requires the use of the appropriate set of markers. The set used here is described below and is shown in Fig 4.

#### Preparation and characterization of the marker ladder for PAGE analysis

**Fig 5:**
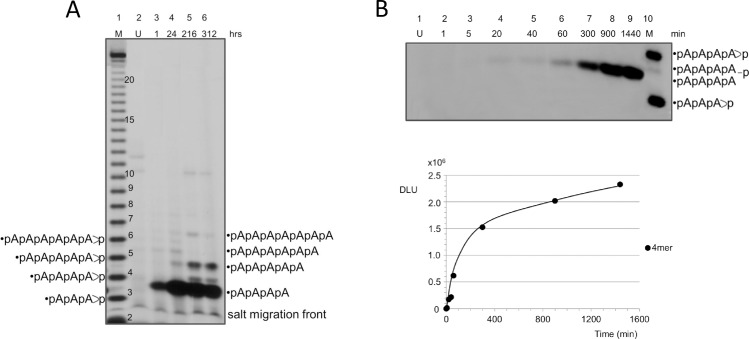
PAGE-analysis of the 3’, 5’ cAMP oligomerization products as a function of time at pH 10.6. Panel A. The material obtained by drying the initial 3’,5’ cAMP solution (pH 10.6) was reacted at 80°C for the indicated times (between 1 and 312 hours, see lanes 3 to 6). U = untreated (see lane 2). Panel B shows a blow-up analysis of the time periods encompassed between 1 and 1440 minutes (lanes from 2 to 9). The plot shows that the reaction rate slows down after 5 hours. Markers (M): formamide-digested 5’-labelled A_24_. For the attribution of the molecular species involved, see the text. Gels with few samples were routinely preferred in order to avoid “gel smiling” effects due to the relatively large concentrations of materials loaded, present in the oligomerization assays. Therefore, the analysis was usually split in small-number groups. Acrylamide = 20%. The salts migration front, below which no attribution is possible, is indicated. The interpolating line in the plot is drawn as a guide to the eye here and in the following figures.

The marker used routinely was a water- or formamide-hydrolyzed A_24_ oligomer. The 24-units oligomer A_24_ was conserved as frozen powder at -20°C and resuspended when needed for terminal labelling and subsequent use as marker ladder in PAGE analyses. 5’-terminal labelling was performed as described in S2 Text. The procedure entails heating at 37°C for 30 minutes in the presence of Mg^2+^. This condition induces partial hydrolysis of the oligomer and provides a partial ladder (Fig 4, Panel A, Lane 1). More extensive and controlled hydrolysis could be achieved treating the A_24_ oligomer with pure formamide (80°C, 16 hrs) after the labelling procedure. The resulting ladder is shown in Fig 4, Panel A, Lane 2. RNA hydrolysis in water is well characterized (ref 13, and references therein) and consists of the cleavage of a phosphoester bond leaving the phosphate bound at 3', which rapidly converts into a cyclic 2' 3' phosphodiester linkage. The latter subsequently opens, leaving a 3'- (or 2'-) bound phosphate group. The double-banded profile in electrophoresis is well documented.[13–15] This second reaction is slower than the first one and in our conditions is detected for monomers, dimers and in traces for trimers and tetramers (as shown). The degradation of RNA oligomers by formamide is similar and was also described in detail (see ref 16 and references therein). Also in this case, the cleavage by formamide leaves the phosphate group of the attacked phosphodiester bond bound at 3', initially in the 2',3' cyclic form (upper band in the band couples, see Fig 4 Panel A). This successively opens (lower band in the band couples) yielding a double-banded profile upon sufficiently long treatment.[16] The bands in the reference ladder are thus made of oligomers bearing two phosphates, one at 5' (introduced by terminal labelling) and one at 3' (from hydrolysis) forming a 2', 3' cycle or, less often, present in open form. The two terminal phosphates (one at 5', one at 3') in the reference oligomers cause a slightly slower gel migration relative to the same-sized neo-synthesized oligomers lacking the phosphate group at 3'.[6–7, 9, 16] These phosphates attributions for the reference ladders and for the neo-synthesized oligomers (as shown in Figs 4 and 5) were validated by MALDI (Figs 2–4). RNA hydrolysis in water is less manoeuvrable and regular than that in formamide (see Fig 4).

#### Conditions for the oligomerization of 3’,5’ cAMP

The oligomerization reaction was analyzed as a function of time, temperature, and pH, exploiting the quantitative accuracy which is an endowed property of the end-labelling/PAGE technique. Overall, the reaction seems to be dependent on the following factors: (i) the time length of the reaction, (ii) temperature and (iii) pH of the reaction mixture before drying. These three parameters concur to form a relatively complex set of interdependent variables, whose untangled description is given below.

#### Time dependence of the 3’, 5’ cAMP oligomerization

First, the PAGE profile of the terminally-labelled products of the reaction (pH 10.6, 80°C) was analyzed in highly concentrated acrylamide gels (20%) in parallel with a reference ladder made of a 5’^32^P-labelled A_24_ oligomer partially digested with formamide [16]. A time period going from 1 min to 312 hours was analyzed (Fig 5, Panel A). A plateau is reached before 24 hours for a population composed of tetramers, while pentamers plateau after 200 hours. Panel B shows a detailed analysis of shorter times, during which no molecules longer than tetramers are formed. In summary, our analysis has shown that synthesis of short oligomers (dimers, trimers) is fast, while further chain elongation requires longer times.

#### Temperature- and pH-dependence of the 3’,5’cAMP oligomerization

**Fig 6:**
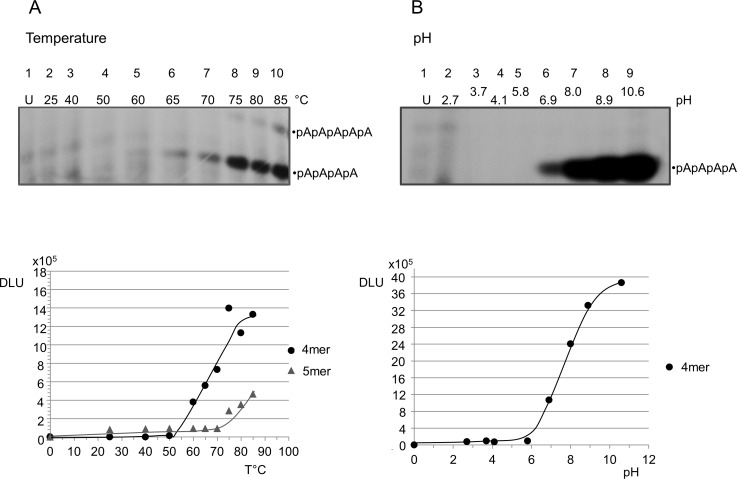
3’, 5’ cAMP oligomerization as a function of temperature and of pH. Panel A: 3’,5’ cAMP oligomerization as a function of temperature, from 25 to 85°C (lanes 2 to 10, respectively), pH 7.0, 24 hrs. Panel B: 3’,5’ cAMP oligomerization as a function of pH, from 2.7 to 10.6 (lanes 2 to 9, respectively). The dry buffered material (for preparation see the Methods section), was reacted at 80°Cfor 6 hrs, purposely in sub-optimal time conditions. U = untreated.

The oligomerization of 3’, 5’ cAMP occurs above a temperature threshold of 60°C (Fig 6, Panel A). The reaction was run at pH 7.0 for 24 hrs, purposely in sub-optimal time and pH conditions in order to avoid possible hydrolytic cleavage of the oligomerization products that could make interpretation of this otherwise very complex, multicomponent reaction mixture even more complicated. The oligomerization is also strongly pH-dependent, it occurs in neutral medium and is stimulated by alkaline conditions, as detailed in Fig 6, panel B.

## Discussion

We have determined the conditions allowing oligomerization of 3',5’ cAMP occurring in the absence of enzymes, inorganic catalysts and templates.

Non-enzymatic polymerization methodologies were developed in the last decade[17–29], extending the in vitro molecular evolutionary studies pioneered by Spiegelman [30]. These polymerizations are based on relatively complex chemistries, involve highly activated precursor monomers (usually phosphorimidazolides) and are well documented and efficient [31]. The relevance of phosphorimidazolides or even of triphosphate nucleotides in early prebiotic scenarios is questionable [32–33], based on the elaborateness of their chemical synthesis and on their relatively high energy content, potentially hampering their accumulation. The synthesis of 2', 3' and 3', 5’ cyclic nucleotides is less demanding and their internal phosphodiester linkage may provide with a moderately high amount of energy to push through transphosphorylation reactions leading to oligonucleotides.

Cyclic nucleotides were pioneerely explored in this perspective four decades ago, presumably for these very reasons, as potential substrates for RNA polymerization. Attention was devoted to the polymerization of the 2', 3’ cyclic form and to its function in the formation of phosphodiester bonds ligating pre-formed oligonucleotides [33–34]. 3', 5’ cyclic purine nucleotides were analyzed only thirty years later [5] showing that 3', 5’ cyclic GMP (hereafter abbreviated as cGMP) affords RNA oligomers in water. More recent studies [6–9] have described this polymerization reaction in detail.

3', 5’ cyclic nucleotides are easily formed in prebiotic conditions [2–3]. Heating nucleosides in the presence of phosphates (i.e., hydroxylapatite or KH_2_PO_4_) causes phosphorylation in every possible position of the ribose (2', 3' or 5'), followed by cyclization at longer reaction times. Hence derives their non-fastidious prebiotic plausibility.

Coupling of the cleavage of the phosphodiester bond in cyclic nucleotides with the contemporaneous formation of a new bond with a correctly positioned neighboring nucleotide, yielding a linear polymer, is thermodynamically favorable. The free energy for phosphodiester bond formation is -5.5 kcal mol^-1^ [35], while the enthalpies of hydrolysis of various 3’,5’ and 2’,3’ cyclic nucleotides vary from -7.7 to -14.1 kcal mol^-1^ [36]. Among all cyclic nucleotides the hydrolysis of 3’,5’ cAMP is the most exothermic, which makes this molecule a dedicated substrate for oligomerization reactions.

**Fig 7:**
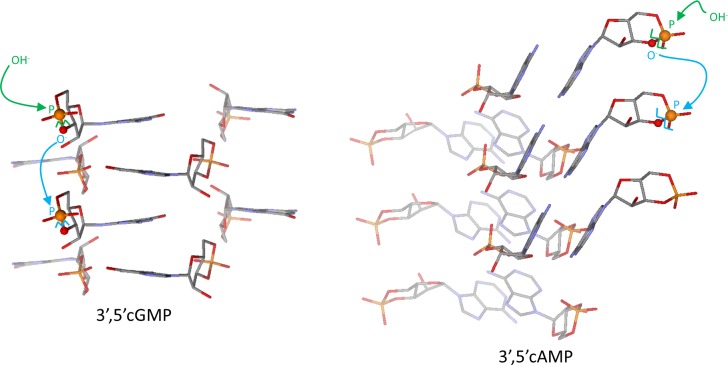
Packing of the cyclic nucleotides in the crystal structure of 3’,5’ cGMP provides with better steric conditions for the transphophorylation leading to oligonucleotides than that of 3’,5’ cAMP [10,11].

**Mechanism of the oligomerization.** In our previous study [9] we have described an anionic ring-opening polymerization scenario for the oligomerization of 3’,5’ cGMP. The reaction is initiated by the base-induced ring-opening of a 3’,5’ cGMP molecule, which produces an O3’-deprotonated nucleotide. We have shown that interbase stacking of guanines in the crystal of 3’,5’ cGMP provides with optimum steric conditions for the attack of the anionic (deprotonated) O3’ of a nucleotide at the phosphorus of the next cyclic nucleotide in the stacked ladder (see Fig 7, left). This step leads to the formation of intermolecular phosphodiester linkages between the cyclic nucleotide precursors and further propagates along the whole stacked architecture. Oligomerization of 3’, 5’ cGMP produces up to 20-mers, whereas the longest oligomer obtained starting from 3’,5’ cAMP was a 4-mer. Looking at the crystal structure of 3’,5’ cAMP and that of 3’,5’cGMP explains the difference in the oligomerization efficiencies.

Fig 7 illustrates the steric conditions for the transphosphorylation reaction leading to oligonucleotides in the crystal of 3’,5’cGMP [10] and 3’,5’cAMP[11]. Main structural parameters derived from the crystal geometries of 3’,5’ cGMP and 3’,5’cAMP are compared in Table 1. Due to the strong preference of guanines to stack the nucleotide packing in the crystal of 3’,5’ cGMP is clearly dominated by interbase stacking interactions. In contrast, the crystal structure of 3’,5’ cAMP is the result of a compromise between interbase H-bonding and stacking. As a result, while the nucleotide units are sufficiently close to each other to react, the in-line attack angles are much less favorable than in the case of 3’,5’ cGMP (see Table 1). In our opinion, this is the reason why the onset of the polymerization of 3’,5’ cAMP requires a noticeably higher temperature (~50–70°C) than that of 3’,5’cGMP (~30°C) [9]. Note that at higher temperatures the thermal motion is more intensive enabling the reactants to sample a more favorable orientation for the in-line attack. For the same reason, oligomerization of 3’,5’ cAMPs leads to considerably shorter products than that of 3’,5’ cGMPs.

**Table 1:** Comparison of the bond distances and angles characterizing the steric conditions for the in-line attack of O3’ at the next phosphate in the crystal structure of 3’,5’ cGMP and 3’,5’ cAMP [10,11].

Structural parameter^a^	3’,5’ cGMP	3’,5’ cAMP
*d*(P_1_…P_2_), Å	7.38	5.82
*d*(O3’_1_…P_2_), Å	6.34	5.49
*<*(O3’_1_-P-O3’_2_), degrees	125.3	93.9

^a^*d*: interatomic distance, *<*: angle. 1 and 2 in the subscripts refer to two adjacent nucleotides in the crystal structure.

Summarizing: 3’,5’ cyclic nucleotides are plausible precursors for an untemplated, non-enzymatic, not-specifically catalyzed RNA origin, because of their conceivable abiotic generation and accumulation, of their controllable reactivity and of the favorable thermodynamics of their polymerization. The reactions described here confirm that also the oligomerization reaction of 3’, 5’ cAMP is coherent with this overall behavior. Nonetheless, the fact that oligomerization of 3',5' cAMPs provide shorter oligonucleotide sequences than that of 3',5' cGMPs whereas it requires somewhat harsher reaction conditions suggests that cyclic GMPs had a better potential to kick-start those simple chemical transformations on the primordial Earth that eventually laid down the foundations of terrestrial life.

## Supporting Information

S1 Fig MALDI-ToF/ToF fragmentation analysis of the non-covalent dimer peak at *m/z* = 657. (PDF)

S1 Text Details of the preparative procedure used to synthesize 3’,5’ cAMP. (PDF)

S2 Text Technical details of the 5’-phosphorylation of the RNA samples used for isotopic labelling. (PDF)
