# Supplementary material for: Non-Enzymatic Oligomerization of 3’, 5’ Cyclic AMP
Source: PLoS One. 2016 Nov 1;11(11):e0165723. doi: 10.1371/journal.pone.0165723 (PMC5089550; doi:10.1371/journal.pone.0165723)
Supplement: S1 Fig — (PDF) [file pone.0165723.s001.pdf]

Fragmentation of the non-covalent cAMP dimer, MS/MS data, negative mode

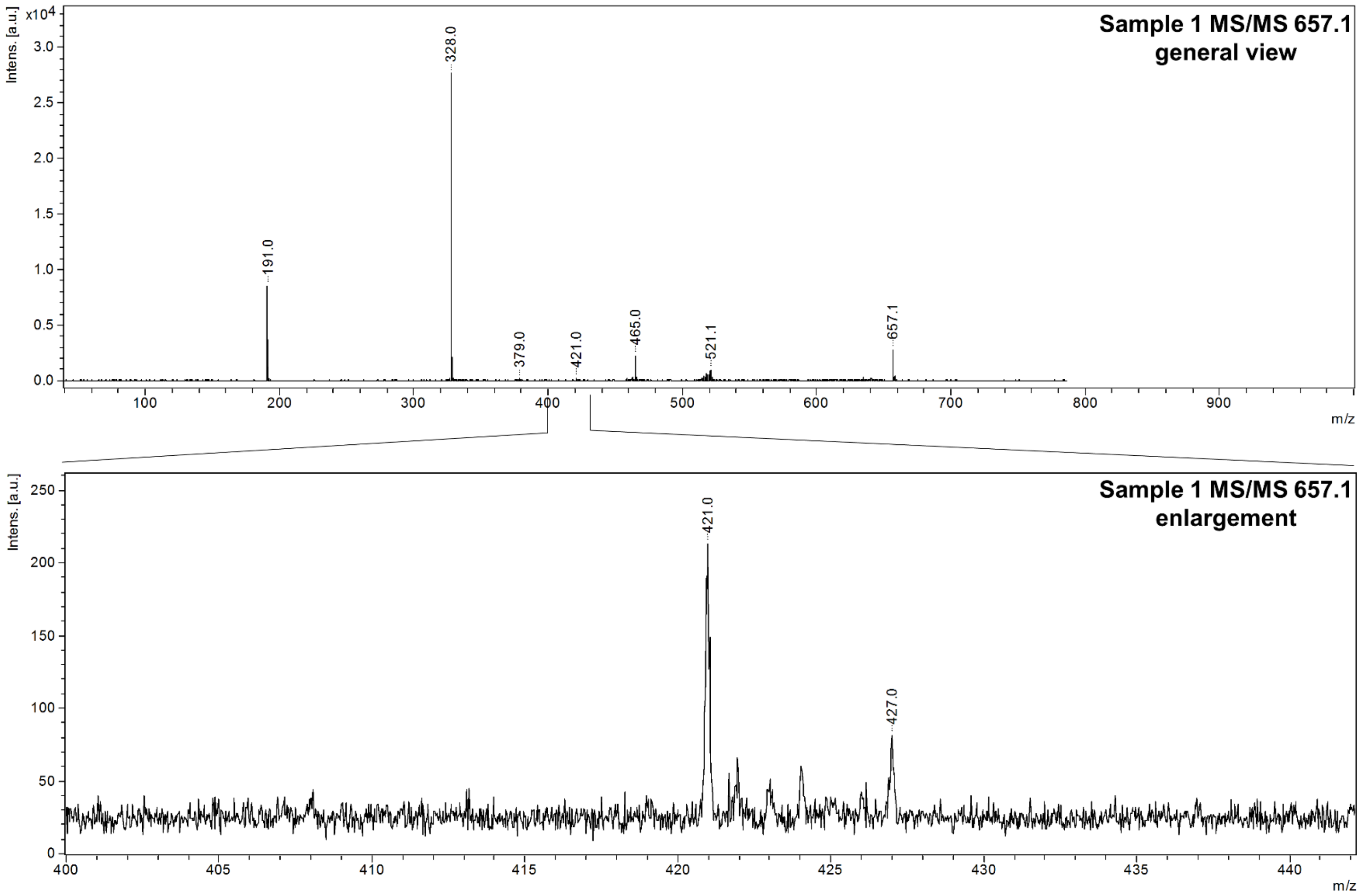

The dominant fragment (*m/z* = 328.0) corresponds to free cAMP.
Signals indicating covalent binding (*m/z* = 408.0, 426.0) were not detected.
