# Supplementary material for: Non-Enzymatic Oligomerization of 3’, 5’ Cyclic AMP
Source: PLoS One. 2016 Nov 1;11(11):e0165723. doi: 10.1371/journal.pone.0165723 (PMC5089550; doi:10.1371/journal.pone.0165723)
Supplement: S1 Text — (PDF) [file pone.0165723.s002.pdf]

SI #1: Preparation of the 3', 5'-cAMP, H^+-form

The process by the manufacturer BioLog consisted in the following: 735 µmol cAMP, Na^+ (BioLog LSI Catalog No. B 004), MW 367.2 g/mol was dissolved in 490 mL deionized H_2O (1.5 mM) and applied to a cation exchange column pre-equilibrated to the H^+-form (5 L 0.5 M HCI followed by 10 L H_2O until neutral) at a flow rate of 5 mL/min. The strong cation exchanger Toyopearl SP-650M (TOSOH Bioscience, Stuttgart, Germany) (binding capacity: 0.15 meq / mL corresponding to a total of 73500 meq) was used. This translated to a 100-fold excess of hydrogen over sodium, if 735 µmol cAMP, Na^+ was applied to the cation exchanger. All product fractions with a concentration higher than 1 mM cAMP, H^+ were pooled and stored at +4°C. The column was regenerated with 5 L 0.5 M HCI and washed with 10 L H_2O to remove all residual Na+ ions. Afterwards, the cAMP, H^+ was applied again to the column to remove all remaining traces of Na+ ions. The resulting product fractions were pooled and filtered through a 0.2 µm membrane. The concentration was adjusted to 1 mM cAMP, H^+ with deionized H_2O and the solution was frozen at -26°C.
