# Supplementary material for: Non-Enzymatic Oligomerization of 3’, 5’ Cyclic AMP
Source: PLoS One. 2016 Nov 1;11(11):e0165723. doi: 10.1371/journal.pone.0165723 (PMC5089550; doi:10.1371/journal.pone.0165723)
Supplement: S2 Text — (PDF) [file pone.0165723.s003.pdf]

SI #2   5' terminal phosphorylation of RNA oligomers

The standard procedure involves phosphorylation by kinase and [γ-^{32}P] ATP. Phosphorylation was carried out by adding 1 µL of T4 Polynucleotide kinase PNK (EC 2.7.1.78, 10 U/µL, New England Biolabs) (Ipswich, MA, USA; # M0201L), 2 µL of 10 × PNK buffer and 0.5 µL [γ-^{32}P]ATP to the neo-polymerized RNA in 20µL, followed by incubation at 37 °C for 30 min. This enzyme catalyzes the transfer and exchange of P_i from the γ-position of ATP to the 5′-OH terminus of polynucleotides, and the removal of 3′-phosphoryl group from 3′-phosphoryl polynucleotides. One unit is defined as the amount of enzyme catalyzing the production of 1 nmol of phosphate to the 5′-OH end of an oligonucleotide from [γ-^{32}P] ATP in 30 min at 37 °C. This procedure typically provides a specific activity of 15000 cpm/pmol.
